# A Pilot Trial to Compare the Long-Term Efficacy of Pulmonary Vein Isolation with High-Power Short-Duration Radiofrequency Versus Laser Energy with Rapid Ablation Mode

**DOI:** 10.3390/jcdd10030098

**Published:** 2023-02-23

**Authors:** Sönke Schildt, Sabine Fredersdorf, Carsten G. Jungbauer, Christian Hauck, Daniel Tarnowski, Kurt Debl, Stefan Neef, Christian Schach, Samuel Sossalla, Lars S. Maier, Ekrem Üçer

**Affiliations:** Internal Medicine II, Cardiology, University Hospital Regensburg, 93053 Regensburg, Germany

**Keywords:** atrial fibrillation, pulmonary vein isolation, high-power short-duration, laser balloon, adenosine

## Abstract

Background: Pulmonary vein (PV) reconnection is the major cause of atrial fibrillation (AF) recurrence after pulmonary vein isolation (PVI). The probability of reconnection is higher if the primary lesion is not sufficiently effective, which can be unmasked with an adenosine provocation test (APT). High-power short-duration radiofrequency energy (HPSD) guided with ablation index (AI) and the third generation of the visually guided laser balloon (VGLB) are new methods for PVI. Methods: A total of 70 participants (35 in each group) who underwent a PVI with either AI-guided HPSD (50 W; AI 500 for the anterior and 400 for the posterior wall, respectively) or VGLB ablation were included in this observational pilot trial. Twenty minutes after each PVI, an APT was performed. The primary endpoint was the event-free survival from AF after three years. Results: A total of 137 (100%) PVs in the HPSD arm and 131 PVs (98.5%) in the VGLB arm were initially successfully isolated (*p* = 0.24). The overall procedure duration was similar in both arms (155 ± 39 in HPSD vs. 175 ± 58 min in VGLB, *p* = 0.191). Fluoroscopy time, left atrial dwelling time and duration from the first to the last ablation were longer in the VGLB arm (23 ± 8 vs. 12 ± 3 min, *p* < 0.001; 157 (111–185) vs. 134 (104–154) min, *p* = 0.049; 92(59–108) vs. 72 (43–85) min, *p* = 0.010). A total of 127 (93%) in the HPSD arm and 126 (95%) PVs in the VGLB arm remained isolated after APT (*p* = 0.34). The primary endpoint was met 1107 ± 68 days after ablation in 71% vs. 66% in the VGLB and HPSD arms, respectively (*p* = 0.65). Conclusions: HPSD and VGLB did not differ with respect to long-term outcome of PVI. A large, randomized study should be conducted to compare clinical outcomes with respect to these new ablation techniques.

## 1. Introduction

Since the pioneering study of Haissaguerre et al., pulmonary vein isolation (PVI) has become the most effective therapy in preventing atrial fibrillation (AF) recurrences [1,2].

Recently, the so-called “high-power short-duration” (HPSD) ablation technique, which causes wider lesions with less depth, has been introduced into the radiofrequency (RF) field with the aim of avoiding collateral damage to surrounding structures [3,4]. Furthermore, the third generation of the visually guided laser balloon (VGLB) ablation system (Heartlight X3) is equipped with a motor, which enables a self-rotation of the laser beam (rapid mode), causing continuous lesion formation around the PV [5]. This allows the application of a higher amount of energy into the tissue, with potentially more efficient lesion formation.

Given these promising new PVI techniques, we designed a prospective observational pilot study to compare the long-term efficacy of PVI with either RF energy using our institutional HPSD strategy, guided by ablation index (AI), or the third generation of VGLB, guided by adenosine provocation testing. The primary endpoint was the event-free survival after three years.

## 2. Methods

In this prospective observational pilot study, patients with symptomatic paroxysmal or persistent AF were enrolled on a weekly basis, such that in one week the patients were treated with a PVI with RF energy and in the other week PVI with VGLB. The indication for PVI was met according to the recommendations of the ESC Guidelines [6]. Written informed consent was obtained from all participants. The study was designed in accordance with the Declaration of Helsinki and approved by the ethical committee of the University of Regensburg.

Exclusion criteria were a history of prior left atrial (LA) ablation; allergic asthma or known allergy to adenosine; presence of left atrial appendage thrombus; LA-diameter > 60 mm; NYHA-IV heart failure symptoms; myocardial infarction within the previous 60 days; unstable angina; any history of mitral valve surgery; uncontrolled bleeding; active infection; renal insufficiency, defined as GFR < 30 mL/min/1.73 m^2^; and severe pulmonary disease.

### 2.1. Ablation Procedure

Every patient underwent cardiac computer tomography before the ablation procedure to assess the anatomy of the left atrium and exclude intracardiac thrombi. The ablation procedure was performed under continued oral anticoagulation with dabigatran 110 mg b.i.d. or phenprocoumon with a target INR of 2.0–3.0 in deep analgosedation. In case of severe obesity or sleep apnea, the procedure was performed under general anesthesia. Unfractionated heparin boluses were administered after transseptal punction to achieve an activated clotting time between 300 and 350 s.

### 2.2. HPSD Ablation

Venous sheaths were placed in both groins (two 7F sheaths in the left femoral vein, two 8.5F SL0 sheaths in the right femoral vein; St. Jude Medical, St. Paul, MN, USA). Double transseptal puncture was performed using an RF needle.

For HPSD and VGLB ablation, a 3D electroanatomical activation map of the left atrium was created with a circular mapping catheter (LassoNav) using the Carto 3 System (Biosense Webster Inc., Diamond Bar, CA, USA), and baseline potentials of each pulmonary vein (PV) were recorded with the EP recording system (Labsytem Pro-EP, Boston Scientific, Marlborough, MA, USA).

**Figure 1:**
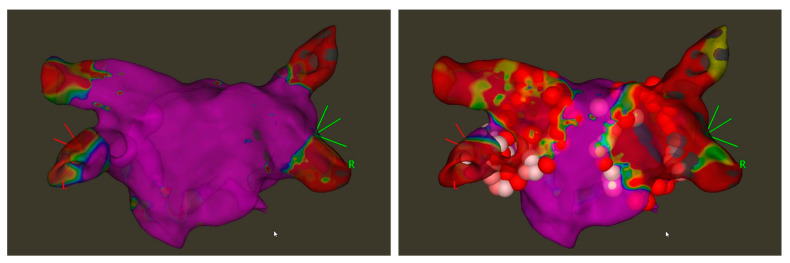
Ablation of the pulmonary vein with high-power short-duration (HPSD), pre- (**left**) and postablation (**right**) electroanatomical voltage map, 0.2–0.5 mV, posterior–anterior view with CARTO.

An ablation catheter capable of measuring contact force was used (Navistar Thermocool Smart Touch SF, Biosense Webster Inc., Diamond Bar, CA, USA). The HPSD ablation technique was used to achieve a circumferential PV isolation in power-controlled ablation mode with 50 W (temperature limit: 43 °C, irrigation rate: 15 mL/min). A minimum contact force of 10 g and a maximum of 20 g were targeted. We aimed to achieve a prespecified ablation index (400 for the posterior and 500 for the anterior wall) at each ablation point, as measured by the AI tool of the CARTO3 Systems. The total RF energy (kWs) was automatically calculated by the EP System (EP Lab Solutions, Boston Scientific) according to the ablation data which it received from the ablation generator. The target interlesion distance was 4–6 mm. A circumferential ablation set around ipsilateral PV was created and assessed with the circular mapping catheter. If ipsilateral veins were isolated with or before the completion of the circumferential lesion set, this was defined as “first-pass isolation”. Otherwise, the gap was detected and ablated until the isolation of all ipsilateral PVs (Figure 1).

### 2.3. VGLB Ablation

**Figure 2:**
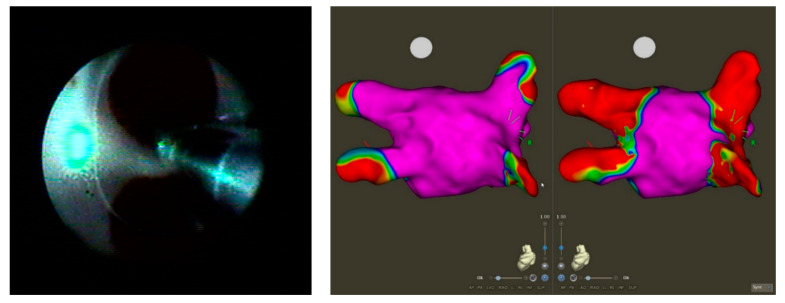
Ablation of the left pulmonary vein with visually guided laser balloon (VGLB) (**left**) and pre- and postablation electroanatomical voltage map, 0.2–0.5 mV, posterior–anterior view (**right**) with CARTO.

The same steps as described above for the HPSD ablation were undertaken until the second transseptal puncture, after which the 8.5 SL0 sheath was exchanged with a 15F laser sheath. An esophageal temperature probe was inserted (CIRCA S-Cath, CIRCA Scientific). Afterwards, the VGLB ablation catheter (Heartlight X3, CardioFocus Inc., Marlborough, MA, USA) was inserted through the 15F sheath and navigated into the PV. The balloon was inflated to achieve complete occlusion of the ostia without moving blood between the balloon and the tissue. In the absence of overlaying blood, an ablation power of 13 W was chosen during rapid mode, striving for a complete ablation circle and isolation after the first encirclement. In areas without optimal occlusion, we changed to manual mode and titrated the power of the VGLB. To avoid gaps between ablation lesions in the manual mode, we applied ablations with a 30–50% overlap using the adjustable 30° aiming arc of the laser beam. Following the first complete encirclement of the individual PV, the VGLB was deflated and the potentials were assessed with the circular mapping catheter. If not isolated, we looked for the gap by leaving the circular catheter in the PV, if possible, or we ablated the suspected area. During ablation of the right-sided PVs, phrenic nerve pacing was performed to prevent severe phrenic nerve injury. Ablation was stopped immediately if the esophageal temperature exceeded 39.5 °C. After PVI, a bipolar voltage map of the left atrium was performed to delineate the isolation line created by the laser ablation (Figure 2).

### 2.4. Adenosine Provocation Test

Each isolated PV underwent an APT 20 to 40 min after isolation. After excluding spontaneous recovery of the PV conduction, an adenosine bolus of 18 mg was administered through the femoral vein. Atrial demand pacing just under the sinus rate was started simultaneously to avoid a sinus arrest which would otherwise lead to the potential underdetection of PV reconnection. Adenosine effect was recognized when at least one *p*-wave was blocked. If no AV-block was induced, adenosine administration was repeated with an increased dose. PV reconnection was diagnosed if the circular mapping catheter revealed PV-potentials in a previously isolated PV. A PV reconnection was classified as temporary if the PV signals disappeared as the effect of adenosine ceased or as permanent if the PV potentials persisted. In either case, repeat ablations were performed in the suspected areas of reconnection. APT was repeated until no reconnection could be induced with adenosine or in case the physician decided to stop further ablations.

### 2.5. Follow-Up

All patients were followed up by the referring primary care physician or cardiologist with Holter monitoring for three-days every third month after the ablation procedure as well as with a physical examination and clinical history during the first year after ablation. If suspicious symptoms occurred, the patient underwent a 12-lead ECG. Clinical follow up was completed by contacting the patient via telephone call. The endpoints of the clinical follow-up were symptomatic recurrence of dysrhythmia and discovery of AF or atypical atrial flutter on a Holter or 12-lead ECG, even if asymptomatic or receiving a second ablation procedure for recurrence of AF or atrial flutter.

### 2.6. Statistical Analysis

Values were distributed as means ± SDs for normally distributed continuous variables, medians and interquartile ranges (IQRs) for skewed distributions (assessed by the Kolmogorov–Smirnov and Shapiro–Wilk tests), and counts and percentages for categorical variables. Statistical analysis was conducted using the Student’s *t*-test (unpaired) for continuous variables with normal distribution and the Mann–Whitney *U* test for variables with non-normal distributions. The chi-square test or Fisher’s exact test was used to compare the categorical variables in different arms. Survival analysis utilized the Kaplan–Meier method; comparison of survival curves was performed with the log-rank method. The coherence of two interval-scaled values was assessed with a linear regression model. Statistical significance was defined as *p* < 0.05. Statistical analysis was performed using SPSS25 (SPSS Inc., Chicago, IL, USA).

## 3. Results

A total of 70 patients were enrolled from August 2019 to December 2020. The baseline characteristics and medication did not differ between the arms (Table 1 and Table 2).

### 3.1. Procedural Characteristics

Procedure duration was similar in both arms (155 ± 39 in HPSD vs. 175 ± 58 min in VGLB, *p* = 0.191; Table 3); however, LA dwelling time and duration from first to last ablation were longer in the VGLB arm (134(104–154) vs. 157(111–185) min, *p* = 0.049, and 72(43–85) vs. 92(59–108) min, *p* = 0.010, respectively). The usage of rapid mode correlated with a decrease in procedure duration (*p* < 0.001, R^2^ = 0.52). Fluoroscopy time, also, was significantly longer in the VGLB arm (12 ± 4 vs. 23 ± 8 min in the HPSD and VGLB arms, respectively).

A total of 270 PV ostia in 70 patients were targeted for isolation (3 patients in the HPSD arm and 7 in the VGLB arm had a left common ostium). A total of 137 (100%) PVs in the HPSD arm and 131 (98.5%) in the VGLB arm were successfully isolated (*p* = 0.242). One patient in the VGLB arm required radiofrequency touch-up ablation for the complete isolation of both left PVs. A total of 60% (42 out of 70) of the ipsilateral PVs in the HPSD arm and 74% (99 out of 133) of the individual PVs in the VGLB arm were isolated after the first ablation circle (Table 4).

### 3.2. Ablation Data for the HPSD Arm

In the HPSD arm, ipsilateral PVs were isolated with an average RF energy of 34(24–37) kWs. The mean time for each ablation point was 13.1 ± 1.27 s, and an average number of 54(37–62) ablation points were needed to achieve circumferential PV isolation. Pure ablation time (time from first to last ablation point) was 13(9–18) min for the left and 9(8–12) min for the right PVs, respectively (*p* = 0.069). From first ablation point to complete isolation, 40(20–51) min for the left and 29(16–34) min for the right PVs were required, respectively (Table 3).

### 3.3. Ablation Data for the VGLB Arm

In the VGLB arm, 32% (43 out of 133) of veins were isolated by first encirclement using rapid mode only. The total laser energy for all four PVs was 14.3(12–16) with a mean power of 11(10–13) W. Rapid mode was used 68% of the time. There was a non-significant trend in successfully isolating the PVs after the first encirclement when only the rapid mode was used (83% with rapid mode only vs. 69% with rapid mode plus manual mode, *p* = 0.059).

In 10 patients, the esophageal temperature exceeded 39.5 °C at the posterior wall, where the laser ablation at that region had to be interrupted.

### 3.4. Adenosine Testing

A total of 137 veins in the HPSD arm and 132 veins in the VGLB arm underwent APT. The time to adenosine testing and the administered adenosine dose did not differ between the arms (25(20–32) vs. 25(20–30) min, *p* = 0.613, and 20.2(18–20) vs. 19.5(18–18) mg, *p* = 0.266, in HPSD and VGLB, respectively; Table 4).

A total of 10 veins (7%) in 8 patients (23%) in the HPSD arm and 6 veins (5%) in 5 patients (14%) in the VLGB arm reconnected after APT. This resulted in an acute durable PVI of 93% (127 veins) vs. 95% (126 veins) in the HPSD and VGLB arms, respectively (*p* = 0.340). The patients’ characteristics with and without reconnected PVs differed only with respect to AF duration (26.6(5–33) vs. 45.9(13–50) months, *p* = 0.037, for negative and positive tested veins, respectively).

In the HPSD arm, 2 LSPVs, 3 RSPVs and 4 RIPVs were reconnected temporarily, whereas only one LIPV was reconnected permanently (Table 5). In the VGLB arm, 2 LSPVs, 2 LIPVs and 1 RIPV were reconnected temporarily, 1 LIPV permanently after APT (Table 6).

In the HPSD and VGLB arms, there were no significant differences in the procedural and ablation data between the APT-positive and -negative PVs.

### 3.5. Reablation after Positive APT

In the HPSD arm, 8 out of 10 veins (7 patients) were reablated after reconnection. Repeat APT was negative in all reisolated PVs. In one patient, 2 PVs’ transient reconnected veins were not reablated due to the operators’ discretion.

In the VGLB arm, 5 out of 6 reconnected PVs (4 patients) were reablated in areas with the earliest PV potentials according to the circular mapping catheter. Three of these reisolated PVs showed no further reconnection after repeat APT. Two reisolated PVs showed recurrent transient reconnection after repeating APT, but no further reablation was performed. In another vein, no additional ablation was performed after positive APT because of native pulmonary vein stenosis according to the preprocedural CT image and resulting suboptimal balloon–tissue contact.

### 3.6. Complications

In one patient in the HPSD arm, two steam pops occurred, detected by a sudden increase in impedance, without further complications. Five patients in the HPSD arm and one patient in the VGLB arm suffered from postinterventional pericarditis (*p* = 0.198).

In one patient without a history of allergic asthma, the APT was terminated prematurely after the testing of the third vein due to severe bronchospasm.

In the VGLB arm, one patient suffered from esophageal ulceration 3 days after PVI without a fistula formation, which was diagnosed with esophagoscopy upon complaints of the patient and was treated with clipping. Another patient suffered from reversible phrenic nerve palsy after ablation with VGLB. There were two events of transient ST-elevation in the inferior leads after insertion of the laser ablation catheter due to air embolism (Table 7).

### 3.7. Long-Term Clinical Follow-Up

**Figure 3:**
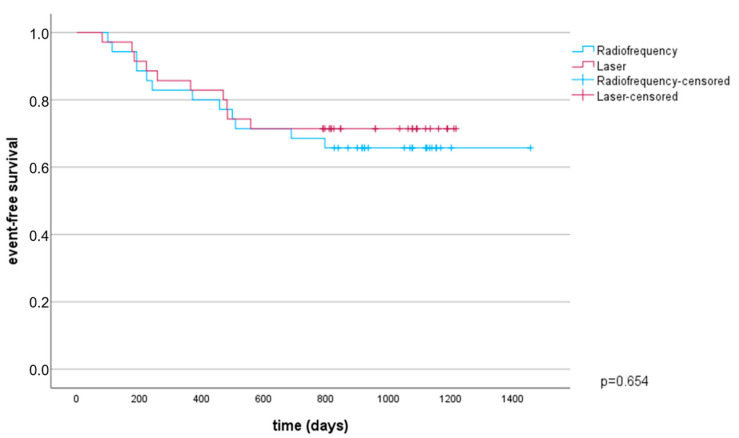
Kaplan-Meier analysis of event-free survival.

The mean follow-up duration after the ablation procedure was 1107 ± 68 days. A total of 10 patients in the VGLB group versus 12 patients in the RF group showed recurrence of atrial fibrillation and/or occurrence of new atypical atrial flutter. Event-free survival at three years of follow-up was 71% in the VGLB group vs. 66% in the RF group, not reaching statistical significance (*p* = 0.65). Only one patient out of five with reconnection in the VGLB group had a recurrence of atrial arrhythmia, whereas six out of eight patients who tested positive in APT tests had a recurrence of AF or atypical atrial flutter (Figure 3).

## 4. Discussion

To the best of our knowledge, this is the first study to compare the acute and long-term efficacy of RF ablation using the HPSD technique and the third generation of VGLB in PVI.

The current study reports, for the first time, that both techniques—AI-guided HPSD RF and the VGLB—seem to be comparable to each other in terms of effectiveness in PVI. On the other hand, the VGLB technique seems to be less safe than the use of RF energy due to the complications that occurred, such as phrenic nerve palsy in one patient and atrio-esophageal ulceration in another. Clinical follow-up showed favorable long-term arrhythmia-free survival over three years with both techniques.

### 4.1. Study Rationale

PV reconnection after initial PVI is one of the main reasons for early AF recurrence [7]. The HPSD ablation has been introduced with the aim of overcoming the difficulties in creating an effective transmural continuous ablation lesion with conventional RF ablation. Studies so far have showed very promising procedural and outcome data for HPSD ablation [8,9].

Given the assumption that transmural and complete PVI reduces PV reconnection and resulting recurrences, there is increasing interest in evaluating the primary ablation effect during the initial PVI procedure [10,11] to validate the primary damage to the tissue. To date, the most effective and best-studied method for this purpose involves performing an APT to unmask dormant PV conduction. The APT is the only currently available method which might be able to test the acute efficacy of new technologies and ablation strategies.

### 4.2. Interpreting the Results

In the current study, both techniques—VGLB and HPSD—showed significantly better acute efficacy with reconnection rates of 5% and 7%, respectively, in comparison to our previous trial [10]. These reconnection rates correlate with other recently published studies, in which very high acute efficacy was shown for the HPSD ablation strategy. Phlips et al., using their CLOSE protocol, achieved a significantly lower reconnection rate compared to conventional RF ablation (3% vs. 18%, respectively; *p* = 0.001) with lower energy settings [12].

### 4.3. Positive Effects of Adding the AI Parameter to HPSD Ablation

There is no consensus about the ablation settings when using HPSD. Even PVI with energy settings of 90 W for 4 s with a special catheter has been shown to be efficient and safe [13]. The duration of ablation varies between 6 and 10 s across different studies. We recently showed an unexpectedly high acute reconnection rate (18%) after PVI with the HPSD strategy using 50 W for 6–10 s [11]. Although in that trial we tried to achieve a CF of more than 10 g and kept the interlesion distance at 4 mm, we attributed this lower-than-expected efficacy of the HPSD strategy to an essential point: the inconsistency of the lesion sets. Since the duration per ablation point was arbitrarily chosen between 6 and 10 s and the combined effects of power, duration, CF and the stability of CF were not considered, the effectiveness of HPSD ablation with fixed duration can be questioned. Accordingly, Chen et al. combined, for the first time, AI with 50 W HPSD ablation and showed the applicability and safety of such a strategy [14]. In our strategy, we used almost the same parameters (the only difference in our trial was an AI of 500 instead of the AI of 550 used in the study by Chen et al. for the anterior wall) but also evaluated acute effectiveness for the first time. Although, the first-pass isolation rate in our study was lower than in the study of Chen et al. (60% vs. 92%), the acute effectiveness of the ablation lesions was very high. Additionally, the duration of ablation per point in our study was comparable to the result of Chen et al. Accordingly, the total procedure duration is shorter than ablation using conventional RF settings.

These new features in RF ablation have advanced this technology, so that the results for acute efficacy are now similar to those for the laser technology, which was not the case in our last study [10]. Meanwhile the advent of the third generation of VGLB has made laser ablation more effective than before, and the strategy of RF ablation with HPSD combined with AI has also gained in effectiveness.

### 4.4. The Influence of the Rapid Mode on the Effectiveness of VGLB Ablation

To facilitate the procedure, the third generation of VGLB was introduced with a self-rotating motor; thus, if optimal balloon–tissue contact is achievable, a complete rotation of the laser beam around the individual PV ostium only takes 2.5 min. Since the laser beam is continuously moving, higher power can be applied to the tissue. Indeed, in our current study, we showed an improved acute effectiveness with a reconnection rate of only 5% (compared to the 11% reconnection rate achieved in our study with the second generation of VGLB) and a shorter procedure and ablation time compared to the former study (175 ± 58 min and 92(59–108) min vs. 232 ± 38 min and 157 ± 34 min, respectively) [10]. In addition, the automated ablation using the rapid mode resulted in lower reconnection rates. In addition, the probability of isolation with the first ablation circle is higher when the rapid mode is used for at least 70% of the ablation circle around the individual PV.

### 4.5. Procedural and Safety Data Comparison

We found significantly higher fluoroscopy times and doses in the VGLB arm, according to our previous findings and other studies [10,15,16]. This was due to the lack of the compatibility between the laser catheter and a 3D mapping system.

Considerable complications, including esophageal ulcer and transient phrenic nerve injury, occurred, respectively, once in the VGLB arm, and there were two air embolisms in combination with the laser sheath, but there also was a non-significant trend towards more postprocedural pericarditis in the HPSD arm.

We used esophageal temperature monitoring only in the VGLB arm, which is obligatory according to the company’s recommendations, but not in the RF arm. This strategy might have led to some subclinical damage in the RF ablation group which might have been overseen. On the other hand, we did not routinely perform esophagoscopy in patients who developed temperature alarm during ablation in the VGLB arm because we immediately stopped ablation in that region. We instructed the patients in both groups about the clinical signs of esophageal damage and performed esophagoscopy in patients with complaints suggestive of esophageal damage.

### 4.6. Influence of the Study Findings on the Previous Study Results

At the end of the day, the clinical outcome, which is freedom from AF after a PVI procedure, is the most important point for the patient. Therefore, clinical-outcome studies comparing different techniques and ablation strategies are needed to find the best ablation method for patients. Since PVI with RF is the most used technique, Dukkipati et al. compared the first generation of laser balloon ablation with PVI using RF and confirmed the noninferiority of PVI with laser balloon compared to PVI with RF energy in terms of arrhythmia-free survival at 12 months and safety [16]. Nevertheless, in that study, PVI was performed either using low-power long-duration RF energy without contact force measurement or with the first generation of the laser balloon system. Since then, the impacts of innovations regarding both ablation methods have not been evaluated. We believe that the new techniques that we used in our study, which were already state-of-the-art PVI techniques, have an influence on acute lesion formation which might lead to better clinical outcomes. The results of our study confirm that both of the new techniques are very effective and comparable to each other regarding acute effectiveness and are acutely more effective than the older RF and laser balloon techniques. Moreover, although not powered to detect clinical outcomes, our study showed similar arrhythmia-free survival rates between the two groups. Moreover, the three-year long-term arrhythmia-free survival rates of 71% in the VGLB group and 66% in the RF group were better than those reported in the first comparison study of Dukkipati et al., which reported a strikingly low arrhythmia-free survival rate in a group of patients solely with paroxysmal AF (61.1% vs. 62.7%). Thus, a new, randomized study should be conducted to compare clinical outcomes with respect to these new ablation techniques.

### 4.7. Limitations

As mentioned above, we planned the study as a pilot study with a small number of patients; accordingly, the limitations of the results due to the small number of patients must be considered. As it was a pilot trial aiming not to determine the clinical outcome at first but the acute effectiveness for each PV, we analyzed the data not by using the number of patients but by using the number of PVs. Knowing that the study was not designed and powered adequately to detect differences in outcomes, we evaluated and presented the long-term clinical outcomes in our pilot study with the aim of setting a basis for further studies with more patients to obtain clarity in terms of safety data, acute efficacy and long-term clinical outcomes with these new-generation ablation techniques.

## 5. Conclusions

The current pilot study shows that AI-guided HPSD ablation via the ablation technique adopted here as well as the third generation of VGLB with a self-rotating laser beam are highly effective tools in PVI and comparable to each other in terms of acute efficacy, as detected by adenosine, and long-term outcomes. Sufficiently powered randomized trials should be performed to compare the long-term clinical outcomes as well as the safety profiles of these techniques.

## Figures and Tables

**Table 1:** Baseline Characteristics.

	HPSD (*n* = 35)	VGLB (*n* = 35)	*p*-Value
Age, years	64 ± 10	61 ± 12	0.220
Male, *n* (%)	20 (57)	20 (57)	1.000
Paroxysmal AF, *n* (%) Persistent AF, *n* (%)	22 (63) 13 (37)	25 (71) 10 (29)	0.445
BMI, kg/m^2^	30 ± 6	29 ± 4	0.170
AF duration, months ^a^	29 ± 34	31 ± 47	0.876
EHRA I-IV	III (3–3)	III (3–4)	0.471
CHA_2_DS_2_VASc	3 (1–4)	2 (1–3)	0.496
Previous cardioversion, *n* (%)	18 (51)	17 (49)	0.811
Previous cavotricuspidale isthmus ablation, *n* (%)	3 (9)	1 (3)	0.614
2D left atrium area in CT, cm^2^	26.0 (20–31)	22.1 (19–24)	0.054
EF < 30%, *n* (EF)	2 (28 ± 0.7%)	0	0.493
Hypertension ^b^, *n* (%)	21 (60)	21 (60)	1.000
Diabetes mellitus, *n* (%)	3 (9)	2 (6)	1.000
History of congestive heart failure, *n* (%)	9 (26)	5 (14)	0.232
Coronary artery disease, *n* (%)	7 (20)	11 (31)	0.274
History of myocardial infarction, *n* (%)	2 (6)	2 (6)	1.000
CABG, *n* (%)	2 (6)	0 (0)	0.493
Valve intervention, *n* (%)	1 (3)	0 (0)	1.000
Hyperlipidemia, *n* (%)	16 (46)	14 (40)	0.629
Stroke, *n* (%)	5 (14)	3 (9)	0.710
Pacemaker, *n* (%)	1 (3)	1 (3)	1.000
ICD, *n* (%)	0 (0)	1 (3)	1.000

^a^ Defined from first documented AF episode to procedure date. ^b^ Defined as blood pressure >140/90 mmHg.

**Table 2:** Medication.

	HPSD (*n* = 35)	VGLB (*n* = 35)	*p*-Value
ACE ^a^ inhibitor or AT1R ^b^ blocker, *n* (%)	21 (60)	22 (63)	0.806
Sacubitril/Valsartan, *n* (%)	2 (6)	0 (6)	0.493
Beta blockers, *n* (%)	30 (86)	27 (77)	0.356
Class IC antiarrhythmics, *n* (%)	5 (14)	6 (17)	0.492
Cass III antiarrhythmics, *n* (%)	3 (9)	1 (3)	0.357
Ca^2+^ channel blockers, non-dihydropyridine type, *n* (%)	1 (3)	1 (3)	1.000
DOACs ^c^, *n* (%)	33 (94)	32 (91)	0.215
Phenprocoumon, *n* (%)	1 (3)	2 (6)	0.555
Diuretics, *n* (%)	13 (37)	7 (20)	0.117
Aldosterone antagonist, *n* (%)	2 (6)	5 (14)	0.428
Ca^2+^ channel blockers, dihydropyridine type, *n* (%)	10 (29)	7 (20)	0.703

^a^ Angiotensin converting enzyme. ^b^ Angiotensin 1 receptor. ^c^ Direct oral anticoagulants.

**Table 3:** Procedure data.

	HPSD (*n* = 35)	VGLB (*n* = 35)	*p*-Value
Procedure duration ^a^, min	155 ± 39	175 ± 58	0.191
LA dwelling time ^b^, min	134 (104–154)	157 (111–185)	**0.049**
Ablation time ^c^, min	72 (43–85)	92 (59–108)	**0.010**
Fluoroscopy dose, cGycm^2^	1302 ± 599	2007 ± 1011	**0.001**
Fluoroscopy duration, min	12 ± 4	23 ± 8	**<0.001**
Ablation energy, kWs	81.0 (50–99)	14.3 (12–16)	**<0.001**

^a^ From groin puncture to sheath removal. ^b^ Time from transseptal puncture to sheath removal out of left atrium. ^c^ Time from first to last ablation.

**Table 4:** Isolation and APT.

	HPSD (*n* = 35)	VGLB (*n* = 35)	*p*-Value
Number of PVs	137	133	
Successful PVI, *n* (%)	137 (100)	131 (98.5)	0.242
First-pass isolation	60% (42 out of 70)	74% (99 out of 133)	**0.034**
Spontaneous reconnection before adenosine testing, *n* (%)	9 (7)	11 (8)	0.594
Time to APT ^d^, min	25 (20–32)	25 (20–30)	0.613
Adenosine doses	20.2 (18–20)	19.5 (18–18)	0.266
PV reconnection after APT, (%)	10 (7)	6 (5)	0.34

^d^ Time from isolation of the individual PV to adenosine administration.

**Table 5:** Ablation data for HPSD.

	APT-Negative (*n* = 127 PV)	APT-Positive (*n* = 10 PV)	*p*-Value
Ablation duration ^a^, min	12 (8–12)	15 (8–22)	0.346
Ablation energy, kWs	33.4 (23–35)	43.2 (26–63)	0.234
Ablation lesion count, *n*	53 (37–58)	67 (48–82)	0.126
Time to APT, min	25 (20–31)	27 (20–35)	0.821
Adenosine dose, mg	19.8 (18–18)	19.2 (18–18)	0.625

^a^ Duration of current application for circumferential ablation.

**Table 6:** Ablation data for VGLB.

	APT-Negative (*n* = 126 PV)	APT-Positive (*n* = 6 PV)	*p*-Value
Ablation duration ^a^, min	6 (3–7)	7 (4–9)	0.254
Ablation energy, kWs	3.7 (2.6–4.3)	4.4 (2.8–6.1)	0.379
Time to APT, min	25 (20–29)	25 (20–36)	0.577
Adenosine dose, mg	19.5 (18–18)	17.0 (17–18)	0.145
Average chosen energy ^b^, W	11.2 (9.6–13)	10.6 (9.0–11.8)	0.362
Fraction of rapid mode, %	69.2 (43–100)	54.8 (30–77)	0.272
First-pass isolation, *n* (%)	98 (78)	1 (17)	**0.003**

^a^ Duration of laser energy application for individual PV isolation. ^b^ Including rapid mode.

**Table 7:** Adverse events.

	HPSD	VGLB	*p*-Value
Postinterventional pericarditis, *n* (%)	5 (14)	1 (3)	0.198
Esophageal ulceration, *n* (%)	0 (0)	1 (3)	1.000
Transient phrenic nerve palsy, *n* (%)	0 (0)	1 (3)	1.000
Steam pops, *n* (%)	1 (3)	0 (0)	1.000
Bronchospasm, *n* (%)	0 (0)	1 (3)	1.000
Stroke, *n* (%)	0 (0)	0 (0)	1.000
Groin hematoma, *n* (%)	0 (0)	0 (0)	1.000
Pericardial tamponade, *n* (%)	0 (0)	0 (0)	1.000
Transient ST-Elevation	0 (0)	2 (6)	0.493

## Data Availability

Not applicable.

## Abbreviations

ACE	Angiotensin converting enzyme
AF	Atrial fibrillation
APT	Adenosine provocation test
BMI	Body mass index
CABG	Coronary artery bypass grafting
CF	Contact force
DOAC	Direct oral anticoagulants
EF	Ejection fraction
EHRA	European Heart Rhythm Association
EP	Electrophysiology
ESC	European Society of Cardiology
GFR	Glomerular filtration rate
HPSD	High-power short-duration
ICD	Implantable cardioverter defibrillator
INR	International normalized ratio
LA	Left atrium
LIPV	Left inferior pulmonary vein
LSPV	Left superior pulmonary vein
NYHA	New York Heart Association
PV	Pulmonary vein(s)
PVI	Pulmonary vein isolation
RF	Radiofrequency
RIPV	Right inferior pulmonary vein
RSPV	Right superior pulmonary vein
